# Microbiota of Sardinian Goat's Milk and Preliminary Characterization of Prevalent LAB Species for Starter or Adjunct Cultures Development

**DOI:** 10.1155/2019/6131404

**Published:** 2019-07-08

**Authors:** Maria Barbara Pisano, Maura Deplano, Maria Elisabetta Fadda, Sofia Cosentino

**Affiliations:** Department of Medical Sciences and Public Health, University of Cagliari, Monserrato 09042, Italy

## Abstract

This work was performed to study the microbiota of raw goat's milk (67 samples) collected in different areas of Sardinia, in order to select autochthonous lactic acid bacteria (LAB) strains for use in goat cheese manufacturing. Total mesophilic bacteria ranged between 10^5^ and 10^7^ cfu/mL; mean counts of* Enterobacteriaceae* did not exceed 4 log cfu/mL whereas those of* E. coli* and coagulase-positive staphylococci were lower than 1.5 and 2 log ufc /ml, respectively. Neither* Salmonella *spp. nor* Listeria monocytogenes *were recovered. The numbers of total LAB were in the range from 10^4^ to 10^7^ cfu/mL and mean yeasts counts varied between 10^3^ and 10^5^ cfu/mL. The most frequently isolated LAB species were* Lactococcus lactis* subsp.* lactis* and* Lactobacillus paracasei*. The presence of* Enterococcus faecium* was also noteworthy. The* in vitro* study of some functional characteristics related to technological properties of the strains belonging to these species allowed to point out some strains possessing good potential for use as adjunct or starter cultures in the production of cheese.

## 1. Introduction

Goat milk production is a crucial contributor to rural economy in many countries, especially in the Mediterranean and Middle East regions. Overall France, Greece, Italy, and Spain produce 49.2% of goat's milk in the Mediterranean region and 9.4% of the world goat's milk (FAOSTAT, 2018). In Italy, the population of dairy goats is reared mainly in extensive or semi-intensive systems. The size of the company is, on average, very small (36 goats/farm), with higher concentrations in Sicily (26.0%) and Sardinia (21.5%), followed by Piedmont, Lombardy, and Veneto, which together represent 22.2% of the total [1].

Due to the growing consumer interest in functional foods, goat's milk has gained popularity mainly because its high digestibility, high nutritional quality, low allergenicity, and potential nutraceutical properties [2, 3]; therefore the production of goat cheeses has shown a growing trend in recent years. Goat milk is very present in Italian supermarkets, both in pasteurized and in UHT versions, but most of it is mainly processed into dairy products for national markets. In Sardinia, goat milk is almost exclusively used for cheeses' production, even if these products are not as famous as Sardinian ewe's cheeses. The diversification of goat milk production is essential but it is dependent on the development of new and valuable products.

Goat milk presents a rich and complex autochthonous microbiota, and its detailed knowledge is essential for the diversification of productions. This microbiota is responsible for the peculiar characteristics presented by fermented goat milk products and is composed by a wide range of microorganisms with different characteristics that can be potentially considered for use by the dairy industry. According to previous surveys, the main components of the autochthonous microbiota are lactic acid bacteria (LAB) belonging to the genera* Lactococcus*,* Lactobacillus*,* Enterococcus*,* Leuconostoc,* and* Streptococcus* [4–6]. LAB are known to produce a number of desirable substances that can improve the flavor, texture, nutritional value, shelf-life, and safety of foods [7], and the majority of the species possess the QPS status [8].

For safeguarding and promoting the traditional Sardinian goat dairy products, information on the microbial diversity of raw milk is the preliminary step to monitor the autochthonous microflora that certainly contribute to the typical organoleptic and quality characteristics of cheeses, in order to search for and select new strains with distinctive patterns of technological properties to be used in cheese production.

The objective of this study was to isolate and identify the dominant microbiota associated with raw goat's milk samples collected from dairy farms located in different areas of Sardinia. The strains belonging to the predominant LAB species were also analyzed by determining some technological properties relevant to their use as adjunct/starter cultures.

## 2. Materials and Methods

### 2.1. Sampling

A total of 67 samples of raw goat's milk collected from dairy farms located in different areas of Sardinia were analyzed. Each sample represented the pooled milk from one single milking of each herd. Samples were transported to the laboratory under refrigeration and analyzed on the same day.

### 2.2. Isolation and Identification of the Strains

Ten milliliters of milk was transferred to a sterile tube containing 90 mL of 2% (w/v) sodium-citrate solution. Decimal dilutions were prepared in sterile solution of 2% (w/v) sodium citrate and plated in duplicate on specific media to enumerate microbial groups.

Total mesophilic bacteria (TMB),* Enterobacteriaceae*,* Escherichia coli, *coagulase-positive staphylococci, presumptive enterococci, lactococci, and lactobacilli, were enumerated and identified according to Pisano et al. [9]. The identification of LAB was confirmed by polymerase chain reaction (PCR) with species-specific primers, as described in the literature [10–14]. Stock cultures were stored at -20°C in De Man Rogosa Sharpe broth (MRS, Microbiol) containing 15% glycerol and propagated three times in MRS broth for activation before use in experiments.

The presence of foodborne pathogens Salmonella spp. and* Listeria monocytogenes* was also investigated. Twenty-five mL of sample was diluted in 225 mL of buffered peptone water, homogenized, and incubated for 18 h at 37°C for the detection of Salmonella spp., and another 25 mL of sample was diluted in Half-fraser broth, homogenized and then incubated for 24 h at 30°C for the detection of* Listeria monocytogenes* according to the ISO methods, respectively [15, 16].

Yeasts were enumerated on potato dextrose agar (PDA, Microbiol) with chloramphenicol (0.01%) after incubation at 25°C for 5 days.

### 2.3. Technological Characteristics of LAB

Several technological properties were studied on 289 LAB isolates. Caseinolytic, lipolytic, and acidifying activity, citrate utilization, and acetoin production were evaluated as reported by Cosentino et al. [17]. Moreover, to evaluate the ability to coagulate milk, tubes with 10 mL of reconstituted skim milk (RSM, Oxoid, Basingstoke, UK) were inoculated with the strains, incubated in a thermostatic water bath at 30°C, and observed after 24 h for milk coagulation, while to assess the activity of the *β*-galactosidase enzyme, one colony of each isolate was emulsified in a tube containing an ONPG (*o*-nitrophenyl- -*β*-d-galactopyranoside) disk (Fluka, Buchs, Switzerland) and 1 mL sterile saline. The tubes were incubated at 37°C, and the yellow staining (positive reaction) was observed within 6 h. All tests were performed in duplicate.

Finally, the LAB strains were screened for the antibacterial activity against the following indicator strains:* Listeria monocytogenes *ATCC 7644,* Escherichia coli *O157:H7 ATCC 35150,* Enterococcus faecalis *ATCC 19433,* Lactobacillus plantarum *DSMZ 20174, and the bacteriocin-sensitive* Lactobacillus sakei *subsp.* sakei *DSMZ 20017, using an agar spot test as reported by Pisano et al. [18].

### 2.4. Statistical Analysis

Microbial counts were calculated as the number of colony-forming units (cfu) per milliliter of sample and reported as log_10_ cfu/mL. Calculations of standard deviations (SD) were also performed. The mean microbial counts of milk samples collected from two geographical areas of Sardinia were analyzed by Student's t-test using the software GraphPad Prism Statistics vs. 3.00. The significant level of test was set at P<0.05.

## 3. Results and Discussion

### 3.1. Isolation and Identification of the Strains

**Figure 1:**
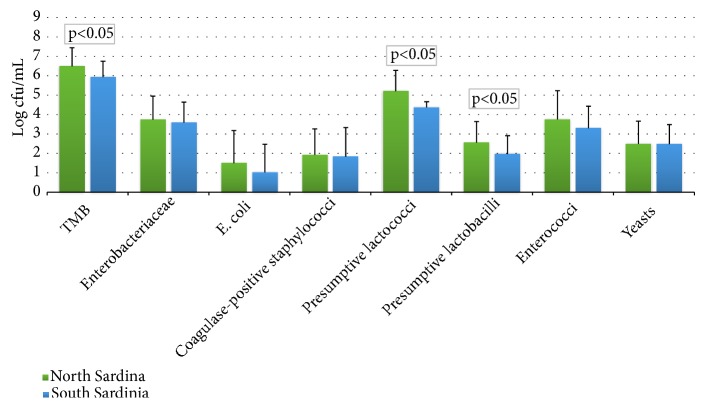
Mean counts ± SD of main microbial groups isolated from raw goat's milk samples collected from dairy farms located in North and South Sardinia.

Figure 1 shows mean values (expressed as log_10_ cfu/mL) and standard deviations for the main microbial groups isolated from the milk samples analyzed. Total mesophilic bacteria (TMB) ranged between 10^5^ and 10^7^ cfu/mL and were significantly higher in samples from north Sardinia. Mean counts of* Enterobacteriaceae* did not exceed 4 log cfu/mL whereas those of* E. coli* and coagulase-positive staphylococci were lower than 1.5 and 2 log cfu /ml, respectively. These values are within the range of those reported in previous works for raw goat's milk used for the production of traditional cheeses in Europe [6, 19]. Although about 50% of milk samples showed total viable counts above the maximum value established by Italian legislation for raw milk intended for further processing (5×10^5^ cfu/mL; Regulation EC 853/2004), it is important to note that LAB accounted for the majority of the microflora, in agreement with the results of the above-mentioned works. However, still there are chances that the microbial load might be enhanced if storage and handling conditions are not appropriate. The presence of* Enterobacteriaceae*,* E. coli,* and coagulase-positive staphylococci in milk is an indicator of unsanitary production and/or improper milking procedures. Millogo et al. [20] pointed out that the differences detected in the microbial load of raw goat's milk samples collected from two farms were related to extrinsic factors such as hygienic conditions during milk handling, season, and geographical location of farms. In our study, all samples were collected in winter and no statistically significant differences were observed for contaminants' counts in samples from north and south Sardinia, while the milking process was performed by hand (by the farmer) in about one-third of the samples; hence the transmission of microorganisms might have occurred.

In relation to the presence of pathogenic species, it is important to note that neither* Salmonella *spp. nor* Listeria monocytogenes *were recovered from milk samples analyzed during the present study.

The numbers of total LAB were in the range from 10^4^ to 10^7^ cfu/mL, and significantly higher (p< 0.05) counts were detected in raw milk samples collected in north Sardinia. Enterococci counts ranged between 10^3^ and 10^5^ cfu/mL. The numbers of presumptive lactococci were higher than those of presumptive lactobacilli. Similar LAB counts were reported by Perin and Nero [5] and de Almeida et al. [3] in Brazilian raw goat's milk samples.

No significant difference was detected in the means of total yeast counts, ranging between 10^3^ and 10^5^ cfu/mL. Even if few studies have been carried out on the total count of yeasts in goat's milk, our data confirmed their low presence [21, 22].

The distribution of LAB species found in raw goat's milk samples is summarized in Table 1.

Of the 308 isolates obtained from M17, MRS, and Kenner Fecal* Streptococcus* (KF) agar plates, 289 were grouped into six genera,* Lactococcus* and* Lactobacillus* being the dominant ones, and 16 species, on the basis of their physiological and biochemical features, while 19 did not grow in subsequent cultures. The majority of LAB isolates were characterized as cocci (195), which have been reported to be the dominant LAB in goat's milk by several authors [4, 5, 23]. The most frequently isolated species was* Lc. lactis* subsp.* lactis*, representing 15.6% of the total isolates, followed by* L. paracasei* which accounted for 14.5%.* Lc. raffinolactis* was the second most frequently recovered species among* Lactococcus* isolates, followed by* Lc. plantarum* and* Lc. lactis* subsp.* cremoris*. Tetrad-forming cocci belonging to* P. pentosaceus* were also recovered, while Leuconostoc and* Streptococcus salivarius *subsp*. thermophilus *were only sporadically isolated. Besides* L. paracasei*, facultatively heterofermentative lactobacilli were represented, in order of frequency, by the species* L. curvatus* and* L. plantarum*, while* L. brevis* was the only species isolated among obligately heterofermentative lactobacilli. The presence of enterococci, mainly represented by* E. faecium *and* E. faecalis*, was also noteworthy. Their recovery in raw milk could be due to faecal contamination, either directly or indirectly through contaminated water sources, milking equipment and bulk storage tanks [24]. Other authors have observed a similar distribution of LAB in raw goat's milk and cheeses [4, 5, 25]. Our results finding* Lc. lactic* subsp.* lactis* as the dominant species in raw goat's milk are in line with those reported by some authors [5, 23], but in contrast with others reporting lactobacilli as the dominant microbiota [4, 26]. The differences in dominant LAB could be attributed to several factors such as different goat breeds, hygienic procedure of milking, and sampling period [26, 27]. The species* L. paracasei* has been previously isolated from both goat milk and dairy farms in Brazil [3] and raw goat milk cheeses [28, 29].

### 3.2. Technological Characterization of LAB

Table 2 shows the results of a preliminary technological characterization of the strains belonging to the predominant LAB species isolated from raw goat's milk, namely,* Lc. lactis* subsp.* lactis*,* E. faecium* and* L. paracasei*. The identification of strains belonging to these species was confirmed by species-specific PCR (data not shown).

As for the acidifying ability, expressed as decrease in pH with respect to the value of noninoculated control milk (pH 6.5) after 24 h of incubation at 30°C in RSM, the majority of* Lc. lactis* subsp.* lactis* strains (25) were found to be good acidifiers, showing a pH drop higher than 2 units after 24 h, while* L. paracasei *and* E. faecium* isolates were slow acid producers, since none or few strains were able to decrease the pH more than 2 units, respectively. The inter- and intraspecies variation in acidification activity were in agreement with the literature [30–32]. Skim milk agar is an effective and rapid medium to detect the extracellular cell-bound proteinases as shown by a clear zone surrounding the colonies. The application of this technique to the 120 strains analyzed showed a good percentage of strains (32%) able to hydrolyze casein, the most active species being* Lc. lactis* subsp.* lactis*. Some caseinolytic activity was also observed in* L. paracasei* strains, as reported by Meng et al. [29]. Proteolytic enzymes play a major role in the fermentation of dairy products [33], since the hydrolysis of milk protein by LAB strains results in an enhanced amount of free amino groups and peptides which are important for microbial growth and as precursor for aroma development during cheeses ripening; however, high proteolytic activity is not always desirable because it can produce large amounts of bitter peptides and other undesirable compounds, or even excessive casein hydrolysis leading to an exceedingly soft final product [17, 34].

None of the strains produced lipolytic reactions on tributyrin agar, in agreement with the results of Meng et al. [29]. Utilization of citrate seemed to be a characteristic of* L. paracasei* strains. A low proportion of these citrate positive strains were also able to produce acetoin. All these properties are confirmed to be strain dependent as they varied significantly among the strains within the same species [4, 17, 35]. The low acidifying and caseinolytic activity as well as the absence of lipolytic activity observed in our lactobacilli and enterococci strains suggest their possible role as adjunct cultures for cheese production, rather than as starters. The majority of* Lc. lactis* subsp.* lactis* isolates (33) were able to coagulate skim milk after 24 h at 30°C revealing, together with their high acidifying ability, their potential as starters in the production of fermented dairy products. In accord with other studies [36, 37], all* Lc. lactis* subsp.* lactis* and* L. paracasei *and the majority of* E. faecium* strains exhibited a strong *β*-galactosidase activity which is the main enzymatic activity responsible for the hydrolysis of lactose. This activity is relevant not only for its technological importance but also from a probiotic perspective, because it can prevent and reduce lactose intolerance.

The antagonistic effect of LAB dairy strains on pathogenic microorganisms could be used for expanding the range of healthful dairy foods. LAB originally isolated from raw milk or artisanal dairy products are probably the best candidate for improving the microbiological safety of these foods, because they are well adapted to the conditions of the substrate.

In this study the 120 strains belonging to the predominant species were preliminarily screened for antimicrobial activity against five indicator strains, including two well recognized foodborne pathogens, by means of an agar spot method (Table 3). Several strains were found to produce an inhibition zone of at least 0.5 mm against the indicators tested. The strongest inhibitory activity was found towards the foodborne pathogens* Listeria monocytogenes *ATCC 7644 and* E. coli* O157:H7 ATCC 35150 by* L. paracasei* and* Lc. lactis* subsp.* lactis* strains.* E. faecium* was more active against* E. faecalis* ATCC 19433,* L. plantarum* DSM 20174, and the bacteriocin-sensitive strain* L. sakei *subsp.* sakei *DSMZ 20017. Our lactococci and lactobacilli strains could therefore be considered potential candidates for control of pathogens in dairy products. Several studies have demonstrated the LAB strains possess considerable inhibitory activity against pathogens and spoilage microorganisms in food (see review by de Sousa and Dias, 2017) [38] by the production of antimicrobial substances, including organic acids, hydrogen peroxide, and bacteriocins [32, 39]. Our strains are currently under investigation to further elucidate their antimicrobial properties.

## 4. Conclusions

Although the characterization of raw goat's milk in terms of LAB composition and technological activity has been carried out by several authors in different parts of the world [3, 4, 6, 19, 23, 26], to the best of our knowledge this is the first study investigating both the biodiversity and technological properties of LAB strains isolated from Sardinian goat's milk samples.

This work was performed to study the microbiota of raw goat's milk collected in different areas of Sardinia, in order to select LAB strains for use as adjunct or starter cultures in the manufacturing of both artisanal and industrial goat's milk cheeses. This local raw milk could serve as source for natural LAB strains, well adapted to milk and cheese environment which can contribute to the safety, quality, and development of typical taste and flavor of the final product.

Predominance of* Lc. lactis* subsp.* lactis* and* L. paracasei *species suggests their important role in manufacturing and ripening of goat's milk cheeses. The* in vitro* study of some functional characteristics related to technological properties allowed to point out some strains belonging to these predominant species possessing good potential for use in the production of cheese. In particular, some* Lc. lactis* subsp.* lactis* strains could be an appropriate starter for goat cheese manufacture, while the* L. paracasei* strains with *β*-galactosidase activity could be used as an adjunct to complement the activities present in the starter and influence flavor development during cheese ripening.

Further studies will be carried out to determine the growth dynamics and behaviour of the selected strains in the cheese environment and their potential probiotic properties.

## Figures and Tables

**Table 1:** Distribution of LAB species in raw goat's milk samples.

Genus	Species	Frequency (%)	N. of strains (289)
*Lactococcus *	*Lc. lactis* subsp. *lactis*	15.6	45
	*Lc. raffinolactis*	6.2	18
	*Lc. plantarum*	5.9	17
	*Lc lactis *subsp. *cremoris*	1.7	5

*Lactobacillus *	*L. paracasei*	14.5	42
	*L. curvatus*	6.9	20
	*L. plantarum*	5.9	17
	*L. brevis*	5.2	15

*Pediococcus *	*P. pentosaceus*	7.3	21

*Leuconostoc *	*Ln. mesenteroides *subsp.* mesenteroides*	3.1	9
	*Ln. mesenteroides *subsp. *dextranicum*	1.7	5

*Streptococcus *	*S. salivarius* subsp. *thermophilus*	2.4	7

*Enterococcus *	*E. faecium*	11.4	33
	*E. faecalis*	7.6	22
	*E. durans*	2.8	8
	*E. avium*	1.7	5

**Table 2:** Functional characteristics of technological interest for prevalent LAB species isolated from goat milk.

		Species (% of positive strains)		
		*Lc. lactis* subsp. *lactis* (no. of strains 45)	*Enterococcus faecium* (no. of strains 33)	*Lb. paracasei* (no. of strains 42)
Casein hydrolysis		37.7	27.2	28.6

Citrate utilization		20	30.3	88

Lipolytic activity		0	0	0

Acetoin production		0	6.1	21.4

Acidifying activity	∆pH (0-1)	37.7	42.4	83.3
	∆pH (1-2)	6.7	51.5	16.7
	∆pH (> 2)	55.5	6.1	0

Milk coagulation		73.3	18.1	21.4

Β-galactosidase activity		100	75.7	100

**Table 3:** Distribution of antibacterial activity in prevalent LAB species isolated from goat milk (% of strains).

	*Lc. lactis* subsp. *lactis* (45 strains)			*E. faecium* (33 strains)			*Lb. paracasei* (42 strains)		
Target strains	0	3-0.5	6-4	0	3-0.5	6-4	0	3-0.5	6-4
	diameter of inhibition zone (mm)								

*L. monocytogenes* ATCC 7644	51.1	20	28.9	63.6	15.2	21.2	0	0	100

*E. coli *O157:H7 ATCC 35150	22.3	37.7	40	33.3	51.5	15.2	42.8	4.8	52.4

*E. faecalis* ATCC 19433	68.9	31.1	0	6	6	88	71.4	28.6	0

*L. plantarum* DSM 20174	84.4	8.9	6.7	0	3	97	78.6	21.4	0

*L. sake*i subsp. *sakei* DSM 20017	89	11	0	0	3	97	66.7	33.3	0

## Data Availability

The data used to support the findings of this study are available from the corresponding author upon request.
